# Alcohol use among amateur sportsmen in Ireland

**DOI:** 10.1186/1756-0500-3-313

**Published:** 2010-11-18

**Authors:** Anne M O'Farrell, Shane PA Allwright, Susan C Kenny, Gerry Roddy, Nazih Eldin

**Affiliations:** 1 Department of Public Health and Primary Care, Trinity College Dublin, Ireland; 2 Department of Public Health, Health Service Executive, Dublin North East Area, Ireland; 3 National Office for Suicide Prevention, Health Service Executive, Dublin, Ireland; 4 Department of Health Promotion, Health Services Executive, Dublin North East Area, Ireland

## Abstract

**Background:**

The objective of this study was to establish baseline data on alcohol consumption patterns, behaviours and harms among amateur sportsmen in the Republic of Ireland.

**Findings:**

The study presents findings from the baseline survey for a cluster randomised controlled trial to evaluate the effectiveness of a community intervention programme to reduce problem alcohol use among a representative sample of Gaelic Athletic Association (GAA) clubs in two counties in the Republic of Ireland. Self reported alcohol use, prevalence of binge drinking, AUDIT scores and alcohol-related harms were assessed in amateur GAA sportsmen aged 16 years and over.

Nine hundred and sixty (960) players completed questionnaires (72% response rate). Mean age was 24.0 years (S.D. 5.2). Of those aged 18 years or over, 75% had post-primary education; most (864, 90%) were current drinkers and 8.2% were regular smokers. The self-reported average yearly alcohol consumption was 12.5 litres. Almost one third (31%) of current drinkers reported drinking over the recommended limit of 21 standard drinks per week and just over half (54.3%) reported drinking 6 or more standard drinks in a row at least once a week (regular binge drinking). Of those who (self) completed the Alcohol Use Disorder Identification Test (AUDIT) questionnaire, three-quarters (74.7%) had a score of 8 or more; 11.5% had a score of 20 or above warranting referral for diagnostic evaluation and treatment. Almost all (87.6%) of the 864 drinkers reported experiencing at least one harm due to their drinking. These alcohol misuse outcomes were higher than those found in a nationally representative sample of males of a similar age. There were strong associations between regular binge drinking and reporting harms such as being in a fight (adjusted odds ratio (OR) 2.02, p < 0.001), missing time from work or college (adjusted OR 1.39, p = 0.04) or being in an accident (adjusted OR 1.78, p = 0.04).

**Conclusions:**

These male amateur sportsmen reported high rates of alcohol consumption and alcohol-related harm.

## Findings

## Background

The link between alcohol consumption and sport is well established and although participation in sport is linked to better physical and mental health, [1,2] there are studies showing that athletes are more likely to engage in binge drinking (drinking six or more standard drinks in one sitting) than their non-athletic peers [3,4]. Evidence suggests that binge drinking and hazardous drinking behaviour may be related to the level of involvement in sports [3,5]. For example, on studying a large cohort of US college students, Weschler et al (1997) found that 60% of students involved in athletics engaged in fortnightly binge drinking compared to 55% of students only partly involved in athletics and 43% of students who had no involvement in athletics [3]. A study by Leichliter et al (1998) reported higher rates of binge drinking among the leaders of sports teams than among team members themselves and team members were more likely to report binge drinking than non-sport team members [4]. A study of New Zealand sportspeople showed that the highest rate of frequent binge drinking was reported by elite provincial level sportspeople (58%), closely followed by elite international/country sportspeople (54%), non-elite (44%) and non-sportspeople (35%) [5]. One study in France showed that compared to peers, sport science students drank less frequently but reported more levels of intoxication [6]. Another study in France found that the level of drinking was high among French adolescents who participated in sports [7]. A study by O'Brien (1993) found that drinking prevalence was high among a small group of sportspeople in Ireland and that any level of alcohol intake appeared to impair aerobic capacity [8].

Ireland is the 2^nd ^highest consumer of alcohol in the EU and binge drinking is common [9-11]. In a national survey of the lifestyle, attitudes and nutrition of people living in Ireland (SLAN survey) 40% of young males (aged 18-29) reported that they binge drink at least once a week and 15% of young males reported that they drink over the recommended weekly alcohol limit of 21 standard drinks [12]. However, there is very little published evidence regarding drinking levels in Irish sportspeople.

The largest amateur sporting organisation in Ireland is the Gaelic Athletic Association (GAA) which has approximately 320,000 playing members and 3,000 clubs, in both urban and rural areas. Games played include hurling, gaelic football and handball. It is estimated that around 8% of the male population play gaelic football and 5% play hurling (a traditional Irish 15-aside stick and ball game), with handball being less common[13]. There is anecdotal evidence that drinking among GAA members is high but hitherto no objective data. The GAA were sufficiently concerned to set up a task force in 2004 to consider how the GAA could promote positive health behaviours within the organisation and the wider community [14]. In response to the task force recommendations, we conducted a cross-sectional survey to assess the extent of the alcohol misuse problem.

## Methods

The study was carried out in two of the four counties in one of the four health board regions in Ireland. This paper presents the baseline data from a community intervention trial (paper in preparation). Logistics precluded us from delivering the intervention to more than 12 clubs in the intervention county. Therefore, a 20% random sample of clubs (12/60) was selected from the intervention county. All clubs (29/29) from the control county were selected. The study populations from both counties were similar at baseline. The only significant differences were: higher proportion in employment, higher proportion of those aged over 18 years completed secondary school (Leaving Certificate) and lower proportion of regular smokers in the intervention group. The study population consisted of all male club players aged 16 years and over. The number of playing members in the selected clubs was approximately 1,325, based on estimates from club managers. There was no upper age limit but few playing members exceeded 40 years of age. Players under 16 years and players who were injured were excluded. Participation in the study was voluntary and confidentiality was assured. Ethical approval was obtained from the Research Ethics Committee of the Faculty of Public Health Medicine of Ireland.

A self-administered questionnaire was used to collect baseline data from the club players. Most of the questionnaires were collected in 2007 (although data collection spanned April 2006 to February 2008). The questionnaire included demographic data (e.g. age, education, employment, playing level) as well as alcohol-related data. Harmful alcohol use was measured using the following:

(a) Alcohol Use Disorder Identification Test (AUDIT) questionnaire [15]. The AUDIT questionnaire consists of 10 items, each scored from 0 to 4. The validity and reliability of the AUDIT have been established[15]. A total AUDIT score of 8 or more is generally considered indicative of harmful alcohol use [16,17]. The World Health Organization (WHO) categorise total AUDIT scores into four risk levels: Zone I, AUDIT score 0-7; Zone II, AUDIT score 8-15; Zone III, AUDIT score 16-19; and Zone IV, AUDIT score 20-40 [17]. Differing levels of intervention are recommended for each risk level (zone): Zone I, alcohol education; Zone II, simple advice; Zone III, simple advice plus brief counselling and continued monitoring; and Zone IV, referral to specialist for diagnostic evaluation and treatment.

(b) A modified version of the Quantity-Frequency scale (QF-scale) [18] was used to measure the yearly alcohol consumption in litres of pure alcohol and to calculate the proportion of those drinking over the recommended weekly limit of 21 standard drinks per week.

(c) Binge drinking was defined for the players of this study as drinking six or more standard drinks in one sitting. This corresponds to the definition used in the SLAN survey (national survey of the lifestyle, attitudes and nutrition of people living in Ireland) [10].

(d) The prevalence of alcohol-related harms was measured using a series of 13 questions that had been used in a national study on the habits of Irish drinkers [11]. These questions have been suggested as useful in measuring alcohol harms in surveys [19].

The data were analysed using STATA and JMP. Pearson's Chi-squared test and Fisher's exact test were used to compare proportions and 95% confidence intervals (CIs) were calculated for proportions of binomial variables and for regression adjusted odds ratios. Multivariate logistic regression analysis was used to analyse the link between alcohol use and related harms. The multivariate regression model was derived using discretionary backward elimination. Biologically relevant variables and variables with bi-variate p values > 0.1 were put into the model but only those that remained significant were retained. Age and education, however, were retained in the models even if not significant as it is standard practice in epidemiological analyses to retain these biologically important confounders.

## Results

Thirty-nine clubs participated in the baseline study (club response rate 39/41, 95.1%) and 960 playing members filled out a usable questionnaire (player response rate 960/1325, 72.5%). Table 1 outlines the demographic and competitive level of the study population. The majority of the participants were in their twenties, single and still living at home. Most (75.4%) of those aged 18 years or over had completed secondary school. Over half of the participants were employed (565, 58.8%) Only 8% (79) were regular smokers. The majority played football (892, 92.9%) with hurling being played by 40% (387) of the participants. Over a third (37.4%) played at senior level.

**Table 1:** Demographic and playing profile of study participants (n = 960)

Demographic characteristics	No. (%)
Mean (S.D.) age in years	24.0 (5.2)

Single	777 (80.9%)

Living with parents	649 (67.6%)

Employed	565 (58.8%)

With Medical Card*	129 (13.4%)

School Leaving Certificate or higher education (in those aged ≥ 18 years)	684/827 (75.4%)

Regular smokers	79 (8.2%)

Playing code	

Hurling	387 (40.3%)

Gaelic football	892 (92.9%)

Dual players	327 (34.0%)

Playing level	

Minor (under 18 years)	93 (9.7%)

Junior	242 (25.2%)

Intermediate	176 (18.3%)

Senior (including inter-county players)	359 (37.4%)

*A Medical Card provides certain health services free of charge but eligibility is means-tested; medical card eligibility is a marker of lower than average household income.

Most of the players (864/960, 90%) were current drinkers. Table 2 presents alcohol use and alcohol-related harms at baseline among the 864 drinkers. The mean yearly alcohol consumption was 12.5 (S.D. 16.8) litres of pure alcohol. The average age at which respondents reported consuming their first alcoholic drink was 15.2 (S.D. 2.8) years. Over half (53.1%) reported having their first alcoholic drink aged 15 years or younger, with just one tenth (10.6%) having their first alcoholic drink aged 18 years or over. Binge drinking status was available for the 864 drinkers; over half (469, 54.3%) reported regular binge drinking.

**Table 2:** Alcohol consumption characteristics of players who were current drinkers (N = 864)

Alcohol consumption characteristics	Total N (%)
Mean (SD) yearly consumption of alcohol (litres of pure alcohol)	12.5 (16.8)

Average (SD) age when first full alcoholic drink was consumed (N = 853)	15.2 years (2.8)

Drinking > recommended weekly limit of 21 standard drink per week	270/864 (31.3%)

Age having first alcoholic drink	

≤ 15 years	540/847 (53.1%)

16-17 years	307/847 (36.3%)

18+ years	90/847 (10.6%)

Binge drinking among current drinkers	

Never binge	68/864 (7.9%)

Infrequent binge (once a month or less)	321/864 (37.1%)

Regular binge (at least once per week)	469/864 (54.3%)

Mean AUDIT score (N = 835)	11.9 (S.D.6.1)

No. (%) AUDIT score ≥ 8	624/835 (74.7%)

**AUDIT Risk Level (Zones)**	

No. (%) AUDIT Zone I, score < 8	211/835 (25.3%)

AUDIT Zone II, score 8-15	411/835 (49.2%)

AUDIT Zone III, score 16-19	117/835(14.0%)

AUDIT Zone IV, score 20-40	96/835(11.5%)

**Alcohol-related harms**	

In last 12 months no. (%) of drinkers (N = 864) who reported: - that drinking harmed their work/studies	265/864 (30.7%)

- felt they should cut down on their drinking	334/864 (38.6%)

- damaged public property because of their drinking	187/864 (21.6%)

- in a fight due to drinking	272/864 (31.5%)

- missed time from work/college due to drinking	368/864 (42.6%)

- in accident due to drinking	168/864 (19.4%)

- been physically sick because of their drinking	570/864 (66.0%)

- felt that drinking harmed their home life/marriage/relationship	141/864 (16.3%)

- felt that they were verbally abusive due to their drinking	257/864 (29.7%)

- felt that drinking harmed your friendship/social life	140/864 (16.2%)

- regretted something said or done due to their drinking	588/864 (68.0%)

- felt that drinking harmed your health	314/864 (36.3%)

- attended A&E due to drinking	100/864 (11.6%)

In last 12 months reported:	
- at least one alcohol-related harm	757/864 (87.6%)
- at least six alcohol-related harms	294/864 (34.0%)

The mean AUDIT score for players who were current drinkers was 11.9 (S.D. 6.1) (Table 2). Almost three-quarters of these respondents had scores ≥8, indicating alcohol use in excess of low risk guidelines; 11.5% (96) were in the highest level of risk (Zone IV) (Table 2).

The proportions of drinkers reporting alcohol-related harms during the past 12 months were high, particularly for acute alcohol-related harms such as being in a fight (31.5%), in an accident (19.4%) or attending an Accident and Emergency Department (A&E) (11.6%) The majority of the respondents reported at least one alcohol-related harm (87.6%) and one third (34.0%) reported at least six harms (Table 2).

Table 3 outlines the factors associated with regular binge drinking. Those who had completed secondary education (i.e. Leaving Certificate or higher education) were almost twice as likely to be binge drinkers as those with a lower level of education. There was a significant negative linear trend for age at having first alcoholic drink and reporting regular binge drinking (p < 0.001). Those who had their first alcoholic drink at 18 years or over were 66% *less *likely to report regular binge drinking than those who had their first alcoholic drink at 15 years or younger (p < 0.001). Being employed and living with parents were also positively associated with regular binge drinking but not significantly when all the relevant factors were in the regression model. Playing level was not associated with regular binge drinking.

**Table 3:** Factors associated with regular binge drinking

	Regular binge drinking (≥ at least once a week)	
	**Odds Ratio (95% CI)**	P-value*

Age (over 18 yrs)	2.66 (1.74-4.07)	p < 0.001

Education (having Leaving Certificate or higher)	1.97 (1.44-2.69)	p < 0.001

Age having 1^st ^drink		
< = 15 years	1.00	
16-17 years	0.55 (0.41-0.74)	P < 0.001
18+ years	0.34 (0.21-0.55)	P < 0.001
Comparing trend all ages with < = 15 years	0.57 (0.46-0.70)	P < 0.001

Employed	1.17 (0.88-1.55)	p = 0.27

Living with parents	1.19 (0.90-1.57)	p = 0.21

Marital status (not being single)	0.97 (0.81-1.16)	P = 0.76

Playing level (low vs. high)	1.03 (0.98-1.19)	P = 0.10

Club bar present	1.07 (0.83-1.39)	P = 0.56

Large club	0.97 (0.73-1.27)	P = 0.82

*Including all factors in regression model.

Regular binge drinking was significantly associated with increased (crude) odds of reporting all of the alcohol harms except for attending A&E (Table 4). For example, those reporting regular binge drinking were more than twice as likely to report being in a fight or that drinking harmed their work/studies (crude odds ratios). After controlling for the factors found in bi-variate analyses to be associated with regular binge drinking (age, age having first alcoholic drink and education), for volume of alcohol consumed, and for the cluster (club) effect, the adjusted odds ratios dropped slightly but remained significant for all but two of the harms.

**Table 4:** Regular binge drinking and reporting alcohol-related harms in the last 12 months

Alcohol-related harm in last 12 months	Crude Odds Ratio (95% CI)	Adjusted Odds ratio* (95% CI)	p-value for adjusted OR
Felt that drinking harmed work/studies	2.54 (1.85-3.47)	1.98 (1.42-2.83)	p < 0.001

Felt should cut down on drinking	2.29 (1.71-3.06)	1.65 (1.19-2.29)	p < 0.01

Damaged public property when drinking	2.21 (1.55-3.16)	1.66 (1.10-2.49)	p < 0.01

In a fight because of drinking	2.20 (1.62-3.00)	2.02 (1.42-2.87)	p < 0.001

Missed time from work/college because of drinking	2.15 (1.62-2.87)	1.39 (1.01-1.92)	P = 0.04

In an accident because of drinking	1.97 (1.36-2.84)	1.78 (1.18-2.86)	p < 0.01

Been physically sick because of drinking	1.94 (1.46-2.60)	1.61 (1.16-2.24)	p < 0.01

Felt that drinking harmed home life/marriage/relationship	1.92 (1.30-2.85)	1.68 (1.08-2.60)	p < 0.02

Verbally abuse when drinking	1.83 (1.34-2.50)	1.59 (1.13-2.25)	p < 0.01

Felt that drinking harmed their friendship/social life	1.75 (1.19-2.58)	1.60 (1.04-2.47)	p = 0.03

Regretted something said when drinking	1.75 (1.30-2.36)	1.44 (1.03-2.02)	P = 0.03

Felt that drinking harmed their health	1.44 (1.08-1.93)	1.12 (0.81-1.54)	p = 0.49

Attended A&E because of drinking	1.32 (0.86-2.04)	1.12 (0.69-1.82)	p = 0.63


*Controlled for age, age having first alcoholic drink and education, volume consumed and cluster (club).

## Discussion

This study found that most (90%) of this group of amateur sportsmen were current drinkers and that self-reported problem alcohol use was high. Over half (54.3%) of the drinkers stated that they binge drink at least once a week which compares unfavourably with the 40% reported in the SLAN survey for males of similar age[12]. Binge drinking prevalence has not been measured among other sportspeople in Ireland, but a sub-group analysis of the national SLAN data showed that highly physically active people were more likely to binge drink than those who reported low or moderate levels of physical activity (Dr. Karen Morgan, personal communication). Research in the United States has shown that those involved in sport were more likely to engage in regular binge drinking than the rest of the population [3,4,20] and the prevalence of regular binge drinking in New Zealand was found to be higher among elite provincial (58%) and international (54%) sportspeople than among non-elite (amateur) sportspeople (44%) and non-sportspeople (35%) [5]. However, the cultural differences in alcohol use between North American, New Zealand and Irish populations make comparison difficult.

The proportion of GAA players reporting alcohol-related harms was higher than that found in the SLAN survey. For example, in the SLAN survey 15% of young men reported being in a fight [12] compared to 31.5% of GAA players. Binge drinking is likely to lead to intoxication and is therefore associated with increased risk of acute physical and social harms. The increased likelihood of harms among regular binge drinkers was independent of the amount consumed. This suggests that the pattern of drinking, as well as the volume consumed, is linked to increased risk of harms.

In the SLAN survey 15% of young males reported that they drink over the recommended weekly limit of 21 standard drinks per week [12]. In this study twice as many (30%) reported drinking over the recommended weekly limit. As drinking over the recommended limit is linked to long term chronic harm such as cancer, cirrhosis of the liver and high blood pressure, [21] this suggests that one in three of the players who are current drinkers may be at increased risk of these alcohol-related health problems in the future.

AUDIT scores were high among this cohort. A quarter of the current drinkers were low risk drinkers (total score < 8, Zone I). However, 14% had AUDIT scores between 16 and 19 (Zone III), a risk level which, according to the WHO guidelines, warrants "simple advice plus brief counselling and continued monitoring"; and 12% had an AUDIT score of 20 or above (Zone IV), indicating the need for referral to a specialist for diagnostic evaluation and possible treatment for alcohol dependence [17]. These alcohol consumption patterns are likely to have both short-term (for those with hazardous and harmful alcohol use patterns) and long-term (for those with dependence symptoms) health and social impacts.

The main strength of this study is that it is the first comprehensive estimate of alcohol use and alcohol-related harms among a representative sample of amateur sportspeople in Ireland. The findings are based on self-report. Given the difficulty of taking blood samples in non clinic-based populations, self-report surveys are commonly used to examine alcohol use and are considered to be valid [22,23]. Interpretation of the demonstrated associations is limited by the cross-sectional nature of the data. As the response rate was high (72.5%), the findings are likely to be generalisable, at least to the study region. The inverse association of alcohol misuse with sport performance and recovery, although of interest, was not investigated here as there is already ample evidence to demonstrate this [24,8,25]. Similar prevalence studies need to be undertaken among athletes participating in other team sports such as rugby, soccer and hockey in order to assess whether they are similar to or differ from GAA players. Similar studies are also required for female sportspeople.

While it might be expected that sportspeople drink less than the general population, evidence suggests the opposite. It is not known why this should be so. It has been suggested that alcohol misuse and alcohol-related harms may be more prevalent in the sporting population due to the age-profile of the cohort (predominantly 18-29 year olds in this study). This age-group are more likely to engage in risk taking behaviour, and are also more likely to be exposed to alcohol marketing [26]. A New Zealand study found that alcohol industry sponsorship in sport was associated with hazardous drinking; sportspeople who received alcohol industry sponsorship had the highest level of hazardous drinking[26]. In addition, sociological research on the collectivity of drinking cultures indicates that people's drinking habits tend to be synchronous with those of their peers. For example, a person living in a low alcohol consumption environment will tend to become a light drinker while an individual exposed to a heavy drinking environment (where drinking is socially sanctioned and encouraged e.g. the sport setting) will tend to become a heavier drinker [27].

Recommendations that may impact at both GAA level and population level need to be considered in light of the collectivity of drinking cultures. These recommendations could include:

(1) Population-wide policies such as increased taxation. Increases in the price of alcohol are particularly effective in reducing consumption in younger people and have a greater impact on more frequent and heavier drinkers [28].

(2) Changes in sponsorship. The alcohol industry is currently a national sponsor of the senior GAA hurling championship. As mentioned above, alcohol industry sponsorship is associated with hazardous drinking [26].

(3) In light of the study findings, the GAA may wish to assess whether its current health promotion programme targeted at preventing substance use within the organisation (Alcohol & Substance Abuse Prevention Programme (ASAP), http://asap.gaa.ie/) needs to be reconfigured and intensified, particularly with regard to club-based substance use policies.

(4) Screening and brief interventions. Recent published research has highlighted the considerable health, social and economic benefits of screening and brief interventions for people at risk of an alcohol-related problem (hazardous drinkers) and those whose health is being damaged by alcohol (harmful drinkers) [29]. The provision of such an intervention in the sports club setting could possibly be undertaken by appropriately trained club coaches.

(5) Prevent the availability of alcohol to those under the legal age for purchasing alcohol. Over half of the players stated that they had their first alcoholic drink at aged 15 years or younger. This requires government level action.

To conclude, this study found that alcohol misuse and alcohol-related harms were higher among male GAA players than in a general population sample of equivalent age but were similar to the levels found among other international athletes. As agreed prior to setting up the study, the study findings have been provided to the GAA to feed into their ongoing alcohol and drug prevention programme (ASAP). Pending information on alcohol use in other team sports in Ireland, the GAA may wish to continue to explore what it can do to change the culture of alcohol misuse among its players.

## Competing interests

The authors declare that they have no competing interests.

## Authors' contributions

The authors of this paper made the following contributions to the paper.

Conception and design, AMOF, SPAA, SCK

Questionnaire design, AMOF, SCK, SPAA

Analysis and interpretation of data, AMOF and SPAA

Have been involved in drafting the manuscript, AMOF, SPAA and SCK

Revising it critically for important intellectual content, AMOF, SPAA and SCK

Have read and given final approval of the version to be published, AMOF, SPAA, SCK, GR and NE.
